# 

**Published:** 1873-12

**Authors:** 


### BOOK NOTICES.

### ROBERT CARTER & BROTHER'S PUBLICATIONS FOR 1874.

No. 530 Broadway, New York.

"*Fanny's Birthday Gift.*" By Joanna H. Matthews, author of the "Bessie Books," etc. 368 pp. 12mo. $1.25.

Also, "*Little Camp*" on Eagle Hill. By the author of the "Wide, Wide World." 429 pp. 12mo. $1.25.

Also, "*Truffle Nephews*," and How they Commenced a New Charity. By the Rev. E. P. Powers, M. A. 270 pp. 12mo. $1.25.

Also, "*Leaves from the Tree of Life.*" By Richard Newton, D. D., author of "Bible Wonders," etc. $1.25.

These volumes are handsomely bound, are suitable presents for the holidays, and, both in manner and matter, like all the books issued by that old and substantial house, are well adapted to amuse, instruct and elevate the family, and all classes will find in them such reading as is well calculated to improve the mind and educate the affections to higher things.

---

### PUBLICATIONS OF HARPER BROTHERS.

"*Farm Ballads*," eleven in all, besides twelve other poems, with twenty illustrations; elegantly bound, and beautifully dedicated "To my Mother." Will Carlton, author.

The first ballad is the far-famed "Betsy and I are Out," followed by "Betsy and I are In." There is so much human nature in these two ballads—so much fun, and so much that is tearful, too—that no one of any appreciation will fail to fall in love with the author, and wish he would go on writing forever.

Also, "*I Go a Fishing.*" 351 pp. 8vo, in Twelve Chapters, on various subjects, by W. C. Prime, whose various books of travel are so entertaining and instructive that any volume he writes is sure to have a multitude of readers.

"*The Bazar Book of Health*," 280 pp. 12mo, deserves a wider appreciation than it will get, because nobody knows who wrote it. The talented author, whoever he is, has no reason to be afraid or ashamed of all the good, and useful, and true things he has written about the Nursery, the Bedroom, Dining Room, Parlor, Library and Kitchen. It richly merits a place in every household. A reader about health must not be placed under the necessity of minutely examining every statement, which must be done unless the writer's name is given. It would be a great benefit to humanity if no attention whatever was paid to anything written in reference to health unless some well known name was attached to it. Health is too precious a thing to be tampered with by following advice given without a name.

---

### LEE & SHEPARD'S PUBLICATIONS.—B ston.

"*Womanhood; Its Sanctities and Fidelities.*" By Isabella Beecher Hooker. 106 pp. 12mo. Paper cover.

Most suggestive of many important practical truths concernin social life.

Also, "*Fireside Saints.*" By DOUGLASS JERROLD. Being *Mr.* Caudle's Breakfast Talk—Mistress Caudle having talked herself to death long ago.

A very amusing book to some; to others, otherwise—being rather calculated to make them laugh on the other side of the mouth.

Also, "*The Turning of the Tide.*" By ELIJAH KELLOGG. Illustrated. 288 pp. 12mo.

Also, "*John Goodson's Legacy.*" By the same author. Illustrated. 304 pp. 12mo.

Also, "*The Young Boat Builder.*" By OLIVER OPTIC. 13 Illustrations. 340 pp. 12mo.

Also, "*Golden Sunbeams.*" Being a collection of New Music for Sabbath Schools, and for the social and home circle. By G. D. F. HODGES and J. H. TENNEY, containing about 150 tunes.

Also, "*The Morning Star.*" By Messrs. HODGES and FOSTER. A collection of Music for Choirs, Singing Schools and Conventions. 384 pp.

"3,000!!! *Words.*" Being a Pronouncing Hand-book of Words often mispronounced.

A half dollar book, of very great value to writers, to the young, and to all who have a laudable ambition for correct pronunciation. The publishers, Messrs. LEE & SHEPARD, Boston, have done the public a service in issuing a volume so practical and so universally needed. It is admirably adapted for the use of schools.

---

"*Yale Lectures on Preaching.*" J. B. FORD & Co., Publishers. 1 vol. 12mo. $1.50. Second series.

The volume is compact with the best of advice, born of experience, and shows an intimate knowledge of the power of the social forces, which goes far to explain the smooth and even working of Mr. Beecher's great organization of Plymouth Church, as well as much of the wonderfully apt and keen treatment of human nature in his own preaching.

J. B. FORD & Co., New York, have published "*A Good Match,*" with suggestions how to make the same, to that vast number of persons who want to wed.

---

"*Sex in Education;*" *or, a Fair Chance for the Girls.* By EDWARD H. CLARK, M. D., Member of the Massachusetts Medical Society, and Professor of Materia Medica in Harvard College. JAMES R. OSGOOD & Co., Boston. 181 pp. 12mo. $1.00.

Plato asks, "Is there anything better in a State than that both women and men be rendered the very best?" Plato answers, "There is not." But this does not prove that Plato believed that boys and girls should be educated together. But the author does, and gives some very strong reasons for his belief. As it is a practical question, seeking solution all over the country, and across the Atlantic, too, this timely volume can be profitably read by the advocates of both sides of the question. Every close observer and student of human nature will have no hesitation in asserting that the co-education of the sexes, like marriage, purifies, elevates and refines both male and female.

YOUNG MEN BOARDING, who are either receiving small wages or being without places, and are waiting for a situation, may find it expedient to obtain accommodations at a low rate for the present. There are worthy families all over the city who are willing to receive one or more boarders at prices ranging from five to twelve dollars a week. Full information can be obtained of Mr. JOHN H. HANKINSON, Secretary of the Boarding House Committee, at the Young Men's Christian Association Building, at the southwest corner of Fourth avenue and 23d street, New York city. The object of the Committee is to place young men—especially those who have recently come to the city to better their fortunes—in respectable, safe and enjoyable surroundings. The charitable and the good of our city are hereby reminded that the Association has increasing and most pressing demands on their treasury, and small amounts are thankfully received, with the guarantee that every penny will be judiciously expended.

NEW CLOTHES.—There is not in the whole United States a larger, more varied or better made assortment of clothing for men and boys, and more suitable for the Holidays, than is found at Baldwin's, the clothier, northeast corner of Broadway and Canal street, New York. The prompt attention and pleasant courtesies always observable at that establishment make a visit of inspection positively delightful, even if no purchases are made; and then again, a copy of *Baldwin's Monthly*, which every visitor can have for the asking, has really more solid instruction and more real fun with the varied reading—moral, religious, philosophical and poetical—than can be found in any octavo magazine in the nation. Baldwin often gives in a single paragraph as much pith—real substance—as is found in an acre of common periodicals.

N. B.—The Editor's office for medical consultation is at 40 Broadway, from 10 A. M. to 3 P. M.; if by letter, address Dr. W. W. HALL, 40 Broadway, New York.

